# Nursing qualifications needed in municipal emergency inpatient units. A qualitative study

**DOI:** 10.1186/s12912-021-00733-w

**Published:** 2021-11-09

**Authors:** Bodil J. Landstad, Torstein Hole, Aasta-Marie Sveino Strand, Marit Kvangarsnes

**Affiliations:** 1 Department of Health Sciences, Mid Sweden University, 831 25 Östersund, Sweden; 2 Levanger Hospital, Nord-Trøndelag Hospital Trust, Kirkegata 2, 7600 Levanger, Norway; 3 Unit of Research, Education and Development, Östersund Hospital, Kyrkgatan 12, 831 50 Östersund, Sweden; 4 Faculty of Medicine and Health Sciences, Norwegian University of Science and Technology, Høgskoleringen 1, 7491 Trondheim, Norway; 5 Møre og Romsdal Hospital Trust, Åsehaugen 1, 6026 Ålesund, Norway; 6 Department of Health Sciences, Norwegian University of Science and Technology, Campus Ålesund, Larsgårdsveien 2, 6009 Ålesund, Norway

**Keywords:** Emergency medicine, nursing education, Primary care, qualification framework, Community of practice

## Abstract

**Background:**

Providing care to older individuals with complex needs and patients with chronic illness is a concern worldwide. In Norway, this situation led to the transfer of responsibility for care and treatment to the municipalities. Providing emergency care at the municipal level – thereby reducing the need for emergency hospital admissions – is part of the Coordination Reform in Norway. This reform from 2012 warrants a reconsideration of which nursing qualifications are needed in the municipalities.

The aim of the study is to explore which professional qualifications nurses need to provide emergency care in municipal emergency inpatient units.

**Method:**

A qualitative design with a hermeneutic approach was employed. Interviewing physicians about nursing qualifications may be considered inappropriate. We believe that this is important for developing knowledge that can strengthen interprofessional cooperation in emergency situations. Three focus groups were conducted. Physicians with experience in municipal emergency inpatient units were interviewed.

**Results:**

We synthesised three themes from the data: (1) broad medical knowledge; (2) advanced clinical skills; and (3) ethical qualifications and a holistic approach. The first theme is about knowledge, the second is about skills, and the third conveys the need for overall competence.

**Conclusions:**

Nurses working in municipal emergency inpatient units need advanced ethical qualifications, which integrate broad medical knowledge, advanced clinical skills and the ability to take a holistic approach. They have a considerable responsibility to work independently and safely in a setting where both the patient and the patient’s family play important roles. Establishing arenas for collaborative practice between physicians and nurses on clinical issues may be a way of strengthening patient safety and nurses’ clinical judgement.

**Supplementary Information:**

The online version contains supplementary material available at 10.1186/s12912-021-00733-w.

## Background

The growing number of elderly individuals with complex needs and chronic illness is a major challenge to the health sector internationally [3, 13, 23, 31]. This situation has led to a transfer of responsibility from the specialist healthcare sector to the primary healthcare sector. In Norway, the Coordination Reform was implemented in 2012. This reform entails a range of legal, financial, professional and organizational policies [10]. One of the tasks in the reform was transferring some of the responsibility for emergency care to the municipalities, as described in the Health and Care Services Act [15].

These new responsibilities increase the demand for qualified healthcare professionals in municipalities. Providing emergency care at the municipal level requires advanced or master’s level qualifications [16]. Admission to a local emergency unit is intended to reduce the need for hospital admissions, and may be considered for a variety of conditions, such as exacerbations of chronic obstructive pulmonary disease or heart failure, or acute conditions like pneumonia or urinary tract infections [14]. The target patient group consists of selected patients with known somatic illnesses, in urgent need of health services. Hospitalization duration is usually less than 4 days. However, a municipal emergency unit demands a strengthening of nursing qualifications and the establishment of new forms of inter-professional collaboration, and continuous nursing attendance [14]. From 2017 the municipalities are also responsible for patients with mental illnesses and substance abuse problems [14].

The aim of this study was to explore which professional qualifications nurses need to provide emergency care in Norwegian municipal emergency inpatient units.

A recent Norwegian study exploring the collaborative practices in municipal emergency inpatient units [20] found that close collaboration between professions was regarded by health personnel as necessary for the development of these services. Physicians are responsible for the medical treatment and play a key role in the way the unit is organized and used. Hence, it would be of interest to identify the nursing qualifications that experienced physicians consider relevant for work in municipal emergency units. The terms competence and qualification are often used synonymously, and therefore, both terms will be used.

The relationship between nursing competence in municipal health services and expected competence in Norwegian policy documents was examined in a previous study [4], and several discrepancies were identified. The study showed that nurses are expected to possess a wide range of competencies, and that many nurses did not possess the advanced level of competence that was expected according to policy documents. Close collaboration between physicians and nurses has been found as crucial for ensuring patient safety. Research has shown that trust among professional groups is considered a prerequisite for quality care [16, 28]. Professional environmental culture, supportive leadership and systemic factors seemed to be crucial to success [28].

The term *qualification* has important contextual aspects, which are emphasized in the research literature [3, 26]. To develop contextual qualifications, nurses need to have insight into what it takes to acquire expertise in the field. This means that in addition to field knowledge, professionals must also understand the historical and social conditions underpinning their work. Ethical considerations should be an integral part of this understanding [26]. Sullivan [26] called attention to the importance of acquiring not only analytical competence and skills, but also an overall general competence. This kind of overall competence can be seen as a kind of practical wisdom or habitus. Sullivan described it as ‘the embodied sense of expert judgement that can do justice to the full range of the profession’s complexity’ (p. 254). The importance for nurses to possess an overall qualification, which includes ethical considerations, is highlighted in the literature [3, 21]. According to Martinsen [21] and Benner [3], only in this way will nurses be able to integrate diverse forms of knowledge, and thereby provide person-oriented professional care. Our study sheds light on the qualifications that Norwegian physicians consider important for nurses to possess in order to meet the complex requirements in municipal emergency inpatient units.

The definition of the term competence varies, and is problematic [8, 25, 29]. Testing competence arose as an alternative to testing intelligence in jobs for which a high level of intelligence was not deemed necessary [29]. Competence, as originally conceived, was intended to describe a low level of ability – and consequently, may be an inappropriate term to use with regard to nursing education. In this study, we instead use the term professional qualification to more fully describe the complexity of the phenomena studied.

In accordance with the European Qualifications Framework (EQF [9];), we have in this study divided professional qualifications into knowledge, skills and general competencies. Knowledge is understanding of theories, facts, principles and procedures in subject areas and/or occupations. Skills are the ability to utilize knowledge to solve problems or tasks, and they include cognitive, practical, creative and communicative skills. General competence is the ability to utilize knowledge and skills in an independent manner in different situations and to show cooperativeness, responsibility and the ability to think critically. This study will provide important insight into what knowledge, skills and general competencies that are expected from nurses in emergency care in municipal emergency inpatient units. This is poorly described in existing guidelines and earlier research.

## Methods

### Research design

A qualitative design with a hermeneutic approach was employed [12, 17]. Taking a hermeneutic approach implies that the text is interpreted with reference to a historical and cultural context. Understanding is developed through interaction and a participatory, dialogic process, which involves interacting with topics considered alien and revealing prejudices, subjectivity, biases and preconceptions [17]. Empathy is an important aspect of interpreting texts.

### Participants and research context

Interviewing physicians about nursing qualifications may be seen inappropriate. We believe it is highly important for developing knowledge that can strengthen interprofessional cooperation in acute situations. Purposive sampling was conducted [24]. The inclusion criteria were physician education and experience with working in a Norwegian municipal emergency inpatient unit. Five men and five women were included in this study. Because we wanted to include physicians who had experience with different ways of organizing municipal emergency inpatient units, physicians from two small towns in Mid-Norway and physicians from seven rural municipalities were invited to take part in the study. The physicians from the two towns shared the same organizational model and cooperated with neighbouring municipalities in organizing their units. The physicians from the rural municipalities worked in emergency units that were set up in local nursing homes.

Three focus groups [19] were conducted with physicians working in emergency inpatient units. The physicians collaborate closely with the nurses and have the medical responsibility in these units. Focus groups were chosen because we wanted to explore physicians’ experiences of nursing qualifications needed in providing care and treatment in municipal emergency inpatient units. Knowledge of physician’s expectations on nursing qualifications are important for developing safety in these health services and to strengthen the interprofessional cooperation. Focus groups stimulated the physicians to discuss experiences in a dynamic interaction that provided insight into a new area of work in the municipal health services.

Two of the groups had three participants, and one group had four participants. Their experience as physicians in local health services ranged from two to 40 years. Five physicians had completed their Norwegian specialization for general practitioners (GPs). This specialization requires 4 years of full-time work at a doctor’s office as a GP. There is also an option to complete part of the specialization at a nursing home or a municipal emergency inpatient unit. In addition, these physicians must work at a hospital for 1 year, complete several weeks of different courses and participate in discussion groups for colleagues. The other physicians were physicians without the GP-specialization. See Table 1 for further details.

**Table 1:** Demographic data

Demographic data	Participants (***N*** = 10)
**Gender**	
Men	5
Women	5
**Education**	
Approved specialists in General Practice (GPs)	5
Physicians with or without other specialities	5
**Experience in primary care** (years)	
1–5	2
6–10	0
11–15	3
16–21	1
> 21	4
**Area**	
Rural	Focus group 1 = 3 physicians
Urban	Focus group 2 and 3 = 7 physicians

The interviews were conducted in municipal institutions, in rooms sheltered from interruptions. The moderator was a researcher and physician with experience in conducting focus groups. One or two researchers assisted during the interviews and took notes related to interactions in the groups [19, 30]. The physicians were asked open ended questions about which nursing qualifications they considered important for nurses to have when working in municipal inpatient units. An interview guide based on the European Qualification Framework (EQF) and the research question was applied. The interview guide developed for this study is provided as Additional File 1.

The physicians spoke about their experiences and their expectations concerning nursing qualifications in the unit. The atmosphere of the focus group interviews was supportive and pleasant, with informants providing rich and clear answers, interacting positively and stimulating each other to share their insights. At the end of the focus group interviews, the researcher who assisted summarized the discussions, and the participants had the opportunity to supplement or correct the summary. The interviews lasted from 60 to 90 min, and they were audio recorded and shortly thereafter transcribed. We considered the data to be saturated when no new information emerged from the interviews. We stopped collecting data when all the themes were saturated [24].

The authors of this article held different positions in relation to the field and had different professional working positions. The interviews were transcribed verbatim (approximately 70 pages in total). Both the transcriptions and the notes taken during the interviews were applied in interpreting the data. The historical and social context of the Coordination Reform constituted an important context during interpretation [30].

In adherence to the EQF, data coding was done according to the three different categories of qualification: knowledge, skills and overall competencies. The data sometimes contained elements from more than one category, and hence, could be coded into different groups. Forthcoming patterns in each group were analysed, and subthemes were synthesized into three main themes expressing the essence of the findings.

The analysis was inspired by two hermeneutic circles: the one between the whole and the parts and the one between preunderstanding and understanding. This refers to that understanding of a text as a whole is based on the understanding of each individual part. The interpretation then becomes a process that oscillates between parts and the overall context. Gadamer [12] emphasises the importance of prejudice for understanding. Prejudice is part of the human horizon of understanding and is as such an important gateway to interpretation and understanding. Separating valid prejudices from invalid ones is a challenge. This was tested in dialogue with knowledge about nursing qualifications that were needed before the Coordination Reform (2012). The interpreter’s pre-understanding was tested with the help of time intervals, consequently, it was changed and adjusted in the light of new experiences [12]. We also included an understanding of our own historicity.

Subthemes evolved from the text and were abstracted into three main themes [11], expressing the type of knowledge, skills and overall competences nurses need in municipal emergency inpatient units. The findings challenged our preunderstanding, and a new insight was developed.

## Results

Ten physicians working in municipal emergency inpatient units in both urban and rural areas of Mid-Norway expressed their understanding of the qualifications that nurses need to meet the complex demands of working in municipal emergency inpatient units. Our interpretation shows that nurses need advanced clinical qualifications. We synthesized our findings into three themes: (1) broad medical knowledge; (2) advanced clinical skills; and (3) ethical qualifications and a holistic approach. The first theme is about knowledge, the second is about skills, and the third conveys the need for overall competence.

### Broad medical knowledge

The physicians were concise in their assessment of what nurses should know. An experienced physician said the following:

‘I would like them to be skilled in managing respiratory problems, heart failure, cardiac arrest, COPD [chronic obstructive pulmonary disease], and indications for and administration of oxygen treatment.’ (Focus group 1, physician 3)

Another emphasized that nurses should be able to work in a systematic way. Nurses’ ability to communicate precisely was also emphasized, and linked to their medical knowledge. The informants noted the importance for those working in an emergency inpatient unit to have broad knowledge of chronic illnesses and acute conditions. The physicians expressed that patients who are admitted to the hospital based on one diagnosis, are often discovered to have complex conditions requiring multiple interventions. In addition, the physicians underscored the importance of knowing and accepting one’s own limitations.

Knowledge of and skills in performing common diagnostic tests were emphasized as essential. For example, one physician stated: ‘We need nurses who can take an electrocardiogram, perform a urine analysis and take a CRP [C-reactive protein], haemoglobin, and other tests that we need in order to follow up the patient’. This topic was highlighted in all of the groups.

In addition, the physicians underlined the fact that patients in emergency inpatient units often were using many medications. Evaluating the patients’ medication lists was therefore considered important and required health-personnel to be knowledgeable in pharmacology.

The importance of nutrition was highlighted. Patients’ nutritional situations should be assessed. One of the most experienced physicians said that she routinely administered a nutrition screening of every patient who was admitted to the emergency inpatient unit. She had experienced that nutrition screening seldom was conducted in specialist healthcare services.

### Advanced clinical skills

Skills in systematic observation and clinical examination were considered important. According to the informants, nurses should be able to detect and identify changes in patients’ conditions. One physician described a typical situation encountered in a municipal emergency inpatient unit as follows:

‘It often looks like a simple problem, but then it develops, and you are dependent on nurses who can identify changes in patients’ conditions and tell when a physician should be called’ (Focus group 2, phycician1).

The informants noted the importance of extensive training in systematic observation and diagnostic work. One of the physicians said the following about this cooperation: ‘When someone comes in with pneumonia, we have a list showing which tests should be administered, possible supplemental tests, and what is to be done the next morning’ (Focus group 3, phycician1). Another physician expanded on this, describing the situation as follows:

‘We have a standardized form showing what tests and measurements should be administered by a nurse. From 1600 hours onwards, there will be no physician to consult in the unit. Then, we have a form with scoring on blood pressure, heart rate, respiration frequency, oxygen saturation and things like that, and in this way, we may detect a possible sepsis’ (Focus group 3, physician 3).

Nurses’ ability to report test results and observations precisely to physicians was considered to be of paramount importance. The informants clearly stated that without trust in this matter, it would be difficult to function as a physician. However, they also reported that there was high variability in knowledge and competence among nurses in this respect.

Due to nurses’ responsibility for administration of medical treatment, the physicians also cited procedures such as intravenous cannulation and giving intravenous infusions as important skills for nurses working in municipal emergency inpatient units. One of the most experienced physicians noted that administering medical treatment required more than medical competence:

‘The most important [thing] is that they feel confident, that they have life experiences that enable them to stand and work in the treatment situation. This is not primarily a medical competence but a human one – not fearing everything that can possibly go wrong but doing what can possibly be done’ (Focus group 2, physician 1).

Communication was emphasized as an essential skill. This included both the ability and willingness of nurses to communicate with colleagues, patients and next of kin, and the ability to communicate clearly while involved in teamwork. A positive attitude towards cooperating with other professions and specialist healthcare services was crucial: ‘We are all part of a team’ (Focus group 1, physician 2).

### Ethical qualifications and a holistic approach

Being confident when dealing with critical and unstable patient situations was highlighted as important. The interviewees used the expression ‘a level head’ to describe this trait (Focus group 3, physician 2).

This expression was used to characterize nurses who remain composed in acute situations and who calmly and efficiently do what is necessary to provide person-centred care. The physicians said that this requires a kind of competence which cannot be taught at universities or university colleges, but is developed through participating in a community of learning and practice.

Having experienced nurses in municipal emergency inpatient units was considered to be of central importance. The interviewed physicians emphasized the importance of continuous learning for nurses working in emergency inpatient units. They suggested visiting hospitals as a way of doing this, however, this had not been implemented in any systematic way.

The physicians valued nurses with positive attitudes towards dealing with professional challenges and developing their own qualifications. At the same time, they conveyed that treatment at the local community level implies that one is more interested in the patient than in this person’s diagnosis. A holistic understanding and advanced ethical qualifications were highlighted. The ability to communicate in a compassionate way with patients and their next of kin about illness and possible death was considered important. Engaging in these kinds of conversations was considered an important aspect in providing treatment and care in accordance with patient preferences. Another important theme highlighted by the informants was the importance of helping patients and their next of kin to understand that hospitalization in an emergency inpatient unit could only be temporary. Health personnel should therefore explain the patient pathway for patients and next of kin.

The interviewed physicians expressed that many patients have multiple diagnoses, which often require physicians and nurses to engage in complex decision making. Inter-professional cooperation and good teamwork, as well as the ability to prioritize and make overall considerations about the best solution for the individual patient, were judged as important. The informants asserted that working at a local community level often meant that the whole family was ‘under treatment’. Adopting a family perspective in care and treatment was considered essential in local community health services.

## Discussion

The increased responsibility of nurses in advanced medical treatment in primary care settings represents a new development internationally, and may require development of new patterns of inter-professional collaboration. According to the interviewed physicians, nurses were responsible for managing the treatment prescribed by physicians, and their qualification in this area was an essential element in the provision of quality services. Our analyses revealed that broad medical knowledge, advanced clinical skills, ethical qualification and a holistic approach are considered important nursing qualifications for providing care in municipal emergency inpatient units. The ability to make overall assessments of situations and adopting a family perspective in care and treatment was considered essential.

The interviewed physicians highlighted the importance of broad medical knowledge when working in local emergency units, as patients often have multiple diagnoses and complex conditions. This need is also highlighted in Norwegian national policy documents (Helse- og omsorgsdepartementet, 2015), and is substantiated by research [4, 16, 28]. An increased demand for nurses with skills in managing a more diverse, complex and acutely vulnerable patient population is also described globally [7]. This demand has led to the creation of advanced practice positions for nurses. Core competencies in advanced practice nursing include clinical care, collaboration, coaching and guidance, leadership and ethical decision-making [6].

In the present study, the importance of nurses’ clinical competence and experience was especially highlighted. Transferring advanced treatment to local community health services increases the demand for advanced clinical skills in this setting. A tremendous shift of responsibility from physicians to nurses in all healthcare settings has occurred over the past 60 years [3]. Benner [3] stated that the early identification of changes in patients’ physical conditions in acute and long-term care facilities is crucial to the safety and well-being of vulnerable patients. This finding is supported by the assessments made by the physicians in the present study. Consequently, sound clinical judgement, the ability to manage treatment, and to precisely and systematically report the patients’ condition to the responsible physician were highlighted by the physicians as important qualifications for nurses to have to ensure patient safety.

Several physicians emphasized the need for screening tools to identify nutritional risk in patients admitted to local emergency units. A Norwegian study identified several obstacles preventing the use of screening tools for nutrition in hospitals [18]. These included poorly defined roles, poor leadership involvement and inadequate nutritional knowledge. Factors that facilitated the use of such tools were good documentation systems, coding for undernutrition and physicians’ commitment to and request for nutrition-related data. The study concluded that nutrition treatment should be aligned with other types of treatment. Defined roles and responsibilities with respect to nutritional screening also seem to be lacking in municipal emergency units.

Other forms of qualifications than those identified in the present study have also been advocated [21]. Martinsen [21] envisioned a more humanistic approach as a necessary supplement to evidence-based practice in care. Martinsen [21] noted the meaning of language in understanding patients and the way they are seen. A standardized practice can block patients’ narratives and prevent alternative understandings of the patient’s care. The interviewed physicians expressed that they wanted nurses with ‘life experience’. This might be what Martinsen [21] highlighted as an ethical dimension of caregiving. She expresses that this always should be present in the interaction with a patient and their family care givers. To support this kind of thinking, health personnel, patients and next of kin together should discuss to identify the best choices from the perspective of the patient. From an educational perspective, preparing nurses to interact with patients in vulnerable phases of illness and life situations requires the inclusion of various forms of knowledge. White Paper 26 (Helse- og omsorgsdepartementet, 2015) on primary healthcare services in Norway called attention to the need for advanced clinical nurses with a master’s level of education and with broad qualifications, which enable them to address the complex situations that arise in local health practice. In this study, the interviewed physicians emphasized the importance of nursing qualifications that cannot be taught at the university. Benner’s [2] description of the qualification process from novice to expert sheds light on learning in practice. Novice nurses learn context-free rules and theories that are functional in their daily practice. Advanced beginners understand the patient situation better, but are still dependent on procedures and need assistance in clinical work. Competent nurses are those who have worked with certain types of patient situations for 2-3 years, and consequently understand their work in a larger context. These nurses can prioritize on the basis of this analytical competence. During the next stage, nurses develop a more general understanding, which enables them to see various patient situations from different perspectives. Nurses with this level of understanding are better able to detect changes in a patient situation at an early stage. Expert nurses are those who are able to work almost intuitively from a deep general assessment of the situation.

According to Benner [2], the journey from novice to expert involves three types of change: (a) a change from the use of abstract principles to the use of concrete experiences, (b) a change from a fragmented understanding of a situation to a more general understanding and (c) a change from observation to participation. Benner’s [2] model of the learning process illustrates that it takes time to develop expert competence in local emergency units.

This study shows that nurses in municipal emergency inpatient units need to have advanced qualifications. Sullivan [26] put forward a clear message to educational institutions: the most important task is ‘to form practitioners who are aware of what it takes to become competent in their chosen domain and equip them with the reflective capacity and motivation to pursue genuine expertise’ (p.253). White Paper 16 [22], titled ‘Quality Culture in Higher Education’, highlights the importance of social and emotional qualities, critical thinking, community conscience and self-reflection. Municipal emergency inpatient units place special demands on communicative and cooperative abilities of their health personnel. Nurses must have compassion [5] and the will to find individual solutions so that they can offer compassionate care to their patients in emergency care.

### Method discussion

This study was reported in accordance to the Consolidated Criteria for Reporting Qualitative Research Guidelines (COREQ) [27]. These findings should not be generalized [24], but it is likely that they can be relevant in other settings where nurses have corresponding responsibilities.

A limitation is that two of the three groups comprised only three physicians. However, these physicians all had long experience and good knowledge about the competence situation in municipalities. Rich data were collected because the physicians showed good insight and engagement in the topic asked.

### Rigour

The different choices made during the research process are explicitly communicated here to make the research process transparent [1, 24]. The EQF was used to ensure that the coding was in accordance with an international understanding of qualifications. Member checking was performed at the end of each focus group interview [24]. All the authors participated in the data analysis. The first author coded the data and made a first draft, which was sent to the co-authors for comments. The co-authors read all the interviews.

## Conclusion

The study gives new insight in what nursing qualifications are needed in municipal emergency inpatient units. The findings show that nurses need advanced ethical qualifications which integrate broad medical knowledge, advanced clinical skills and the ability to take a holistic approach. They have a considerable responsibility to work independently and safely in a setting where both the patient and the patient’s family play important roles. This requires that nurses have advanced clinical qualifications so that they can contribute to patient safety and quality in health services.

### Clinical implications

The primary health care sector is increasingly becoming responsible for care and treatment of elderly patients with complex health conditions, warranting a close examination of which nursing qualifications are necessary to fulfil this new role. Nurses working in municipal emergency inpatient units should possess broad medical knowledge, skills in communication and treatment management, and a capacity for continuous learning. User participation and the family perspective are important considerations.

Establishing arenas for collaborative practice between physicians and nurses on clinical issues may be a way of strengthening patient safety and nurses’ clinical judgement. Professionals in the education of nurses and physicians should cooperate to prepare their students for new patterns in inter-professional work with respect to the transition of emergency care to municipalities.

## Supplementary Information

**Additional file 1.**

## Data Availability

The dataset generated and/or analyzed during the current study is not publicly available due to confidentiality. It can be obtained from the corresponding author on reasonable request.

## Abbreviations

COPD: Chronic Obstructive Pulmonary Disease
COREQ: Consolidated Criteria for Reporting Qualitative Research Guidelines
CRP: C-Reactive Protein
EQF: European Qualifications Framework
GP: General Practitioner
WHO: World Health Organisation
