# Supplementary material for: Nursing qualifications needed in municipal emergency inpatient units. A qualitative study
Source: BMC Nurs. 2021 Nov 9;20:223. doi: 10.1186/s12912-021-00733-w (PMC8576997; doi:10.1186/s12912-021-00733-w)
Supplement: Supplementary file 1 — Additional file 1. [file 12912_2021_733_MOESM1_ESM.docx]

Interview guide

1. How have you experienced municipal emergency inpatient units?
2. What knowledge is important for nurses in municipal emergency inpatient units?
3. What skills do nurses in municipal emergency inpatient units need?
4. What overall competences do you consider relevant?
5. Is there anything else you wish to say?
